# Alloying Effect on Transformation Strain and Martensitic Transformation Temperature of Ti-Based Alloys from Ab Initio Calculations

**DOI:** 10.3390/ma16176069

**Published:** 2023-09-04

**Authors:** Honglin Fang, Xingge Xu, Hualei Zhang, Qiaoyan Sun, Jun Sun

**Affiliations:** 1 State Key Laboratory for Mechanical Behavior of Materials, Frontier Institute of Science and Technology, Xi’an Jiaotong University, Xi’an 710049, China; hlfang@stu.xjtu.edu.cn (H.F.); xingge970213@stu.xjtu.edu.cn (X.X.); junsun@mail.xjtu.edu.cn (J.S.); 2 Key Laboratory of Shaanxi Province for Craniofacial Precision Medicine Research, College of Stomatology, Xi’an Jiaotong University, Xi’an 710049, China

**Keywords:** Ti alloys, *β → α*″ phase transition, lattice strain, first-principles calculations, elastic properties

## Abstract

The accurate prediction of alloying effects on the martensitic transition temperature (*M*_s_) is still a big challenge. To investigate the composition-dependent lattice deformation strain and the *M*_s_ upon the *β* to *α*″ phase transition, we calculate the total energies and transformation strains for two selected Ti−Nb−Al and Ti−Nb−Ta ternaries employing a first-principles method. The adopted approach accurately estimates the alloying effect on lattice strain and the *M*_s_ by comparing it with the available measurements. The largest elongation and the largest compression due to the lattice strain occur along ±[011]*_β_* and ±[100]*_β_*, respectively. As compared to the overestimation of the *M*_s_ from existing empirical relationships, an improved *M*_s_ estimation can be realized using our proposed empirical relation by associating the measured *M*_s_ with the energy difference between the *β* and *α*″ phases. There is a satisfactory agreement between the predicted and measured *M*_s_, implying that the proposed empirical relation could accurately describe the coupling alloying effect on *M*_s_. Both Al and Ta strongly decrease the *M*_s_, which is in line with the available observations. A correlation between the *M*_s_ and elastic modulus, *C*_44_, is found, implying that elastic moduli may be regarded as a prefactor of composition-dependent *M*_s_. This work sheds deep light on precisely and directly predicting the *M*_s_ of Ti-containing alloys from the first-principles method.

## 1. Introduction

Ti-based alloys are good potential candidates in biomaterials due to their excellent biocompatibility and low elastic modulus. TiNi-based shape memory alloys (SMAs) have been successfully used in orthodontic archwires [1] and bone implants [2]. To avoid the hypersensitivity and toxicity of the Ni element, it is, therefore, necessary to develop Ni-free biomedical SMAs [3,4,5,6,7,8,9,10,11,12,13,14,15,16,17,18,19,20,21,22,23,24,25,26,27,28,29,30,31,32]. Particularly, TiNb-based SMAs have attracted great attention because of their excellent shape memory effect and superelasticity. There has been broad investigation on TiNb-based alloys, such as Ti−Nb−Al [4,5,6], Ti−Nb−Ta [7,8,9,10,11], Ti−Nb−Zr [12,13,14], Ti−Nb−Sn [15,16,17,18], Ti−Nb−Zr−Ta [19,20], and Ti−Nb−Zr−Sn [21,22,23].

The phase transformation temperature of pure Ti from the *α* phase (hexagonal close-packed (hcp)) to *β* phase (body centered-cubic (bcc)) is 1154 K [3]. When the content of *β*-stabilizing elements is low, the hcp martensite (*α*′) and orthorhombic martensite (*α*″) can be created from the *β* austenite phase by high-speed cooling [33]. Additionally, the hexagonal *ω* phase can be generated from the *β* phase via severe plastic deformation [34,35,36] and from the *α*′ phase under the drive of high-temperature torsion [33,35,37,38,39,40]. The *ω* phase is detrimental to the shape memory effect and superelasticity of martensite. In this work, we mainly focus on the phase transition of *β* to *α*″. To some extent, alloying elements can tune the mechanical properties and phase transformation temperature of Ti alloys. Upon adding *β*-stabilized elements, such as Nb and Mo, Nb can stabilize the *β* phase and lower the transformation temperature of Ti alloys [41,42]. Both experiments [5,7,12,13,15,23,41,43,44] and first-principles calculations [45,46,47,48] have extensively investigated TiNb-based SMAs. From an experimental point of view, the alloying of Al [5], Zr [44], Sn [15], and Ta [7] into Ti−Nb alloys can affect superelasticity, shape memory effect, and martensite transition temperature (*M*_s_). It has been reported that the *M*_s_ linearly decreases by about 40, 30, 38, and 150 K with 1 at. % increases in Nb [43], Ta [7], Zr [44], and Sn [15] additions, respectively. Ti−Nb22−*x*Ta (*x* = 2–8, in at. %) ternary alloys display a stable shape memory effect and superelasticity at room temperature (RT). Note that the compositions mentioned here are given in at. %. Since the *ω* phase has an adverse effect on the shape memory effect, it is found that the doping of Al can greatly hinder *ω* formation [26,27]. From a theoretical point of view, Neelakantan et al. [45] proposed a thermodynamics-based model to estimate the *M*_s_ of Ti alloys. They created a linear relation between the predicted *M*_s_ and molybdenum equivalent ([Mo]_eq_), suggesting that the predicted *M*_s_ increases with decreasing [Mo]_eq_. Minami et al. [47] and Sun et al. [48] correlated the *M*_s_ and the energy difference between the *β* and *α*″ phases in Ti−Nb binaries. Moreover, *C*′ and *C*_44_ are regarded as predictors of composition-dependent *M*_s_ in Ni_2_MnGa-based SMAs [49] and Ti−Ni binary alloys [50]. Since the overestimation of the existing predicted *M*_s_ compared to the measurements, there is still a big challenge to directly predict the *M*_s_ through first-principles methods.

Another challenge is to enhance the recoverable strain of the material while reducing the *M*_s_ meantime. The recoverable strain of Ti−Nb SMAs is around 3% [41] and is smaller than that of Ti−Ni superelastic alloys [51]. Both recoverable strain and the *M*_s_ in TiNb-based SMAs [7,9,52] increase with decreasing Nb content. Namely, there is a conflict between high deformation strain and low *M*_s_ in TiNb-based SMAs [48]. The contradiction between the strain and the *M*_s_ can be alleviated by alloying [53], owing to the coupling alloying effects. Compared to the strong Nb effect [41] on the transformation strains of Ti-Nb binaries, Al [4,5,6,54] and Ta [7,9] elements have a weaker influence on transformation strains in Ti−Nb−X ternary alloys. For instance, a maximum recoverable strain of 4.7% appears if doping 3 at. % Al into Ti−24Nb−Al alloys [4]. Alloying Ta can reduce the transformation strains along [011]*_β_* on average by 0.28%/1 at. % [9]. However, the alloying effect of Nb [41], Al [4,5,6,54], and Ta [7] show similar magnitude orders on the *M*_s_. The measured *M*_s_ decreases by 40, 40, and 30 K, with an increase of 1 at. % Nb, Al, and Ta, respectively.

To overcome low recoverable strain and high *M*_s_ trade-off, Sun et al. [48] calculated the lattice deformation strains using a first-principles method. Meanwhile, they correlated calculated energy differences between the *β* and *α*″ phases and the measured *M*_s_ for Ti−Nb binaries and, thus, estimated an empirical relationship [48] to predict the *M*_s_ of multi-principal element alloys. It has been found that the trade-off is somehow improved or even broken by alloying. Such empirical relationships [48] can roughly describe the composition dependence of the *M*_s_ [48]; however, it overestimates the measured *M*_s_ to some extent. Aiming to accurately predict the *M*_s_, here, we take Ti−Nb−Al and Ti−Nb−Ta as representative alloy systems to search for a better correlation between the *M*_s_ and the change in energy. In the present work, theoretical equilibrium lattice constants, lattice strains, and total energies in the *β → α*″ phase transformation for Ti−22Nb−(0–10)Al and Ti−22Nb−(0–10)Ta ternary alloys are calculated using a first-principles method. A pseudobinary approach is ‘used to associate the measured *M*_s_ with the calculated energy difference between the *β* and *α*″ phases. A satisfied agreement is acquired between the predicted and measured *M*_s_. Based on our proposed relationships considering the coupling effect of alloying, the estimated *M*_s_ can accurately reproduce the composition dependence of measured *M*_s_. Furthermore, it is found that elastic moduli, *C*_44_ and *C*′*,* may be regarded as prefactors of the composition-dependent *M*_s_. This work can precisely predict alloying effects on the *M*_s_ of Ti-based alloys from first-principles calculations. The computational methods are described in detail in Section 2. The results and discussion of the alloying effect on the lattice deformation strain, stereographic projections of the lattice strains, and comparisons between the predicted and measured *M*_s_ for Ti-based alloys are shown in Section 3. We put forward a conclusion in Section 4.

## 2. Methodology

The total energies were calculated using the first-principles exact muffin-tin orbitals (EMTO) [55] method. The self-consistent calculations were performed using the Perdew–Burke–Ernzerhof (PBE) generalized gradient approximation [56]. The Kohn–Sham equations were solved with scalar-relativistic approximation and soft-core approximation. To integrate the valence states below the Fermi level, Green’s function was calculated for 16 complex energy points. The basis sets included the *s*, *p*, *d*, and *f* orbitals in EMTO. The alloys considered here were nonmagnetic. The random distribution in a solid solution was described by the coherent-potential approximation (CPA) [55], implying that the degree of the atomic order of a solid solution is treated in a completely disordered way. To process the electrostatic correction to the CPA, the screened impurity model [57] was used with a screening parameter of 0.6. The *k* point meshes were carefully chosen to describe the tiny changes in energy for different phases. Hence, the used *k* point meshes were 25 × 25 × 25 and 11 × 11 × 11 for the *β* and *α*″ phases in the irreducible Brillouin zone. The theoretical equilibrium lattice constant was determined by fitting the total energies of nine different atomic volumes based on a Morse equation of states.

Based on the crystallographic relationship among the *β*, *α*″, and *α* phases [9,41,48], the *α*″ phase is equivalent to the *β* phase if *b*/*a* = *c*/*a* = $\sqrt{2}$ and shuffle *y* = 0, while the *α*″ phase turns into the *α* phase if *b*/*a* = $\sqrt{3}$ and *y*/*b* = 1/6. Consequently, four variable parameters, including the Winger–Seitz radius (*w*, in Bohr), the axis ratios of *c*/*a* and *b*/*a*, and shuffle *y*, dominate the total energy upon the *β → α*″ phase transition. It is found that a small volume difference produces a relatively tiny energy difference of less than 0.2 mRy for a given phase [58]. Therefore, to reduce the complexity of structural optimization, we ignore the influence induced by volume on different phases when calculating the total energy and consider total energy as functions of *b*/*a*, *c*/*a*, and *y*. In the present study, the range of the *b*/*a* is 1.40–1.75, and the interval of the *c*/*a* is 1.40–1.70. The interval of *y* is set from 0 to 1/6*b* for every *b*/*a* and *c*/*a*. Spline interpolation is chosen to find the equilibrium shuffle *y* in each group of *b*/*a* and *c*/*a*. Then, two-dimensional cubic interpolation is used to determine the equilibrium *b*/*a* and *c*/*a*.

The cubic austenite phase has three independent elastic constants [59,60]: *C*_11_ (the uniaxial deformation along [001]*_β_*), *C*_12_ (the shear stress at (110)*_β_* along [1$\bar{1}$0]*_β_*), and *C*_44_ (the shear deformation along (100)*_β_*). The mechanical stability criteria for a cubic crystal are *C*_44_ > 0, *C*_11_ + 2*C*_12_ > 0, and *C*_11_ − *C*_12_ > 0. The standard technique [61,62,63,64] was used to calculate the *C*_11_, *C*_12_, and *C*_44_. Theoretical values of *C*′ and *C*_44_ were calculated by using the EMTO-CPA method [65]. The *C*_11_ and *C*_12_ were computed from bulk modulus, *B* = 1/3 (*C*_11_ + 2*C*_12_), and tetragonal shear constant, *C*′ = (*C*_11_ − *C*_12_)/2. The *k* point meshes were assigned as 29 × 29 × 29 for the *β* phase.

## 3. Results and Discussion

Transformation strain principally affects shape recovery strain. The martensitic transformation strain depends on lattice strain and lattice correspondence [9,41,48,53,66]. The lattice correspondence between the *β* austenite phase and *α*″ orthorhombic martensite phase is displayed in Figure S1 and can be described, as below:

$${\left[100\right]}_{\beta }-{\left[100\right]}_{\alpha \prime \prime },\;\;{\left[010\right]}_{\beta }-{\frac{1}{2}\left[01\bar{1}\right]}_{\alpha \prime \prime },\;{\left[001\right]}_{\beta }-{\frac{1}{2}[011]}_{\alpha \prime \prime }.$$

(1) $$\begin{array}{c} {\left[100\right]}_{\alpha \prime \prime }-{\left[100\right]}_{\beta },{\left[010\right]}_{\alpha \prime \prime }-{\left[011\right]}_{\beta },\;{\left[001\right]}_{\alpha \prime \prime }-{\left[0\bar{1}1\right]}_{\beta }. \end{array}$$

The lattice deformation strains *η*_1_, *η*_2_, and *η*_3_ [9,41,48,53,66] along the three principal axes of [100]*_β_*, [011]*_β_*, and [0$\bar{1}$1]*_β_* are written as follows:

(2) $$\eta_{1}=\frac{a^{\prime }-a_{\beta }}{a_{\beta }},\;\eta_{2}=\frac{b^{\prime }-\sqrt{2}a_{\beta }}{\sqrt{2}a_{\beta }},\;\eta_{3}=\frac{c^{\prime }-\sqrt{2}a_{\beta }}{\sqrt{2}a_{\beta }}.$$

where *a*′, *b*′, and *c*′ represent the lattice constants of the *α*″ phase and *a_β_* is the lattice parameter of *β* phase.

**Figure 1:**
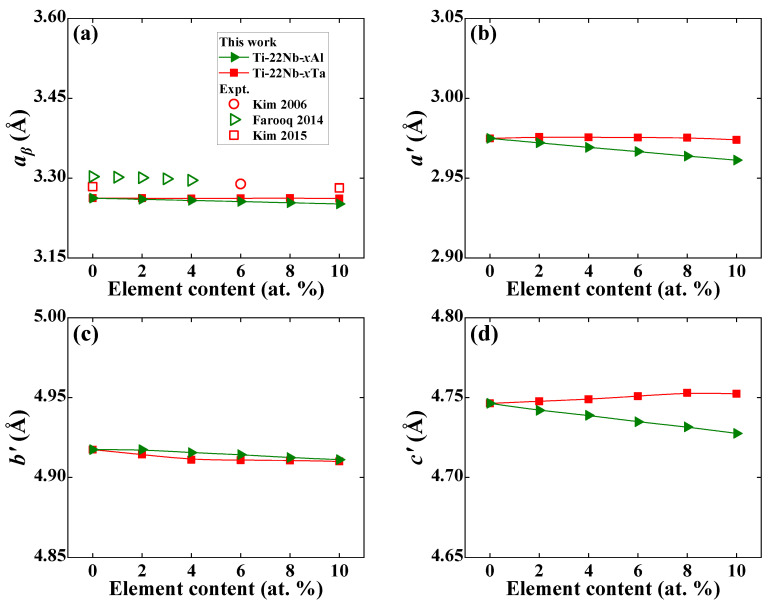
Theoretical equilibrium lattice constants of (**a**) *a_β_* in *β* phase and (**b**) *a*′, (**c**) *b*′, (**d**) *c*′ in *α*″ phase for Ti−22Nb−*x*X (*x* = 0–10, in at. %; X = Al, Ta) as a function of alloying elements, compared with the available experimental values [4,9,11].

Figure 1 shows the present theoretical equilibrium lattice constants of Ti−22Nb−*x*X (*x* = 0–10, X = Al, Ta) in the *β* and *α*″ phases, compared with the available experimental data [4,9,11] in the *β* phase. Theoretical lattice constants from our static calculations are generally smaller than the experimental values. The partial reason may come from ignoring the thermal expansion caused by temperature effects. Alternatively, the deviation between calculation and measurement partially contributes to the different alloy compositions in our selected Ti−22Nb−Al and the measured Ti−24Nb−Al. However, the same composition dependence of the lattice constant appears for both theoretical calculations and available measurements. For example, the lattice constant *a_β_* of Ti−Nb−Al ternaries decreases about 1.07 × 10^−3^ Å/1 at. % with an increase in Al, which is consistent with the available experimental decrement of 1.71 × 10^−3^ Å/1 at. % [4] and 1.9 × 10^−3^ Å/1 at. % [6]. For Ti−Nb−Ta ternaries, *a_β_* keeps almost constant at around 3.26 Å, which is in line with the previous first-principles calculations [67].

Like the decreasing *a_β_* of Ti−Nb−Al, the lattice constants *a*′, *b*′, and *c*′ of Ti−Nb−Al in the *α*″ phase also linearly decrease with increasing Al content. The *a*′, *b*′, and *c*′ of Ti−Nb−Al reduce by 1.37 × 10^−3^ Å, 6.76 × 10^−4^ Å, and 1.85 × 10^−3^ Å with a 1 at. % increase in Al, respectively. The situation becomes complex for *α*″ Ti−Nb−Ta ternaries. The *a*′ of Ti−Nb−Ta is insensitive to Ta content, but the *b*’ first decreases and then increases with increasing Ta content, while the *c*′ shows a linear increasing trend. Such complicated composition dependence agrees with the available measurement [9] and first-principles calculations [67], although the changes in *a*′, *b*′, and *c*′ for a given Ti−Nb−Ta system are somehow scattered [9,67]. For example, the *a*′, *b*′, and *c*′ for Ti−37.5Nb−(12.5, 18.75)Ta alloys [67] increase as Ta content increases, while the *a*′ (*b*′ and *c*′) increases (decrease) with alloying Ta into Ti−(14–18)Nb−(0–10)Ta alloys [9], differing from the theoretical trends in Figure 1b–d. This deviation may come from different Nb and Ta contents and different experimental processes.

**Figure 2:**
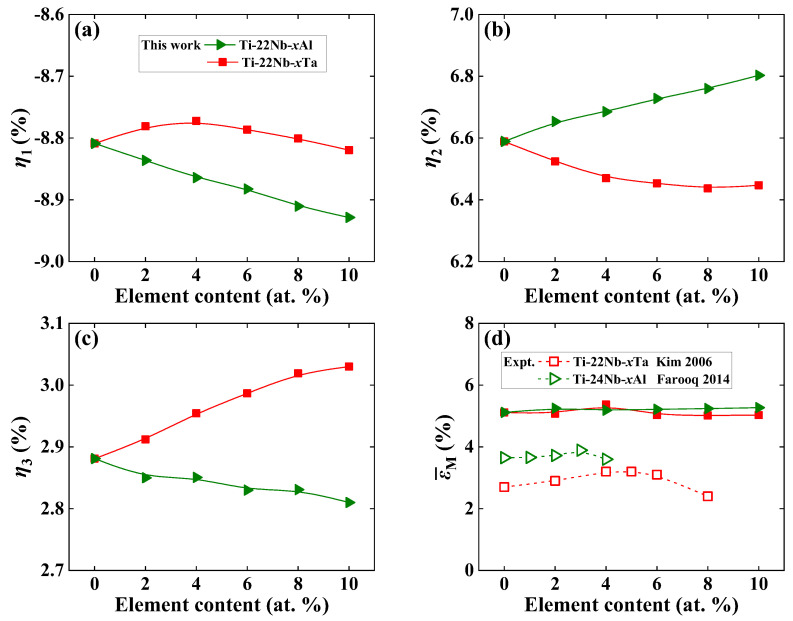
Theoretically predicted lattice deformation strains (**a**) *η*_1_, (**b**) *η*_2_, (**c**) *η*_3_, and (**d**) the predicted average maximum transformation strains, ${\bar{\varepsilon }}_{M}$, of Ti−22Nb−*x*X (*x* = 0–10, in at. %; X = Al, Ta). For comparison, the available experimental maximum recovery strains [4,7] are also displayed.

In Figure 2a–c, we display the lattice deformation strains *η*_1_, *η*_2_, and *η*_3_ of Ti−22Nb−*x*X (*x* = 0–10, in at. %; X = Al, Ta) using Equation (2). It is generally accepted that there is a positive correlation between lattice strain and recoverable strain in SMAs [32,48]. From Figure 2, it can be observed that *η*_1_ is negative, while *η*_2_ is positive, indicating that the martensitic contracts (expands) the lattice along the [100]*_β_* ([011]*_β_*) direction. This finding is consistent with the available measurements in Ti−Nb−Ta [9]. Note that *η*_1_ (*η*_3_) is the largest (smallest) among all three deformation strains in Ti−Nb−Al and Ti−Nb−Ta ternaries. For Ti−Nb−Al alloys, the absolute magnitudes of *η*_1_ and *η*_2_ (*η*_3_) increase (decreases) with increasing Al content, relative to the increase (decrease) in lattice strain. Additionally, the doping of Al produces different variations in lattice strains, which is different from previous theoretical [48] and measured [41,43] observations in Ti−Nb binary alloys. Unlike Ti−Nb−Al, the composition dependence of *η*_1_, *η*_2_, and *η*_3_ of Ti−Nb−Ta ternary alloys is opposite to that of Ti−Nb−Al. The absolute magnitudes of both *η*_1_ and *η*_2_ (*η*_3_) reduce (increases) with increasing Ta content, implying a decrease (increase) in lattice strain. It is found that the present predicted *η*_2_ increases (decreases) with alloying Al (Ta), which is in line with former first-principles calculations [47]. Note that theoretical *a_β_* in the *β* phase is smaller than the available experimental one by an overall error of 1.2%, as shown in Figure 1. The calculated *a*′ in the *α*″ phase is also underestimated, but theoretical *b*′ and *c*′ are rather close to the measurements. Therefore, the absolute magnitudes of *η*_1_, *η*_2_, and *η*_3_ calculated by Equation (2) are all larger than the available measurements [6,9,11]. The measured *η*_1_, *η*_2_, and *η*_3_ in Ti−24Nb−3Al [6] are −2.96%, 2.98%, and −0.04%, respectively. The measured *η*_1_, *η*_2_, and *η*_3_ in Ti−17Nb−10Ta [9] are −2.28%, 2.56%, and −0.38%, respectively, and the measured *η*_1_, *η*_2_, and *η*_3_ in Ti−22Nb−6Ta [11] are −2.07%, 2.47%, and −0.44%, respectively.

Kim et al. [41] proposed an approach to calculate the maximum transformation strain ($\varepsilon_{M}^{i}$) along a certain orientation and the average maximum transformation strain (${\bar{\varepsilon }}_{M}$) for a polycrystal with randomly distributed grains. Following Kim’s approach [41], the lattice distortion matrix (**T**) during the *β → α*″ phase transformation relative to the coordinates of the *β* phase can be illustrated, as in Equation (3):

(3) $$T=\left[\begin{array}{ccc} \frac{a’}{a_{\beta }} & 0 & 0\\ 0 & \frac{b\prime +c\prime }{2\sqrt{2}a_{\beta }} & \frac{b\prime -c\prime }{2\sqrt{2}a_{\beta }}\\ 0 & \frac{b\prime -c\prime }{2\sqrt{2}a_{\beta }} & \frac{b\prime +c\prime }{2\sqrt{2}a_{\beta }} \end{array}\right]$$

Supposing a stochastic vector, $\vec{x}$, in the *β* phase is transformed to $\vec{x\prime }$ in the *α*″ phase due to martensitic transition, the maximum transformation strain, $\varepsilon_{M}^{i}$, along every orientation, can be evaluated, as in Equation (4):

(4) $$\varepsilon_{M}^{i}=\frac{\left|\vec{x^{\prime }}\right|-\left|\vec{x}\right|}{\left|\vec{x}\right|},\vec{x^{\prime }}=T\vec{x}.$$

Kim’s approach has been successfully applied to predict the $\varepsilon_{M}^{i}$ and ${\bar{\varepsilon }}_{M}$ of Ti-Nb binaries [41,68] and TiNb-based ternaries [9,66]. To distinguish different strains, Figure S2 in the Supplementary Materials displays the relationship of the lattice deformation strains (*η*_1_, *η*_2_, and *η*_3_), the maximum transformation strain ($\varepsilon_{M}^{i}$), and the average maximum transformation strain (${\bar{\varepsilon }}_{M}$). According to Equations (3) and (4), 57 representative orientations (i.e., the vertex and the midpoint of the edge at each standard stereographic triangle, as shown in Figure S3a) located in the standard stereographic circle are chosen to exhibit the stereographic projections of lattice strains along (100)*_β_* and (001)*_β_*, respectively.

Based on Kim’s [41] approach, as shown in Equations (3) and (4), we first choose 13 representative orientations located in the [001]−[011]−[111] standard stereographic triangle (shown in the Figure S3b) and calculate the $\varepsilon_{M}^{i}$ along these orientations. Consequently, the predicted ${\bar{\varepsilon }}_{M}$ can be obtained by spline interpolation of the $\varepsilon_{M}^{i}$. Thus, we compare the calculated ${\bar{\varepsilon }}_{M}$ for both Ti−Nb−Al and Ti−Nb−Ta alloys in Figure 2d, along with the available measured maximum recovery strains [4,7] for comparison. According to Figure 2d, the ${\bar{\varepsilon }}_{M}$ predicted by Equations (3) and (4) for Ti−Nb−Al and Ti−Nb−Ta ternaries are higher than the experimental values. This is partially from the underestimation of theoretical *a_β_* and *a*′ in the *β* and *α*″ phase. Furthermore, the measured recovered strains also depend on the tensile strains, which are limited due to the increasing remaining plastic strain. Despite the fact that the predicted ${\bar{\varepsilon }}_{M}$ for both alloys are somehow overestimated compared to those of the experimental counterparts, the composition dependence of theoretical ${\bar{\varepsilon }}_{M}$ reproduces the measurements [4,7]. From Figure 2d, it can be observed that the theoretical ${\bar{\varepsilon }}_{M}$ first weakly increases and then decreases with an increase in Al and Ta contents, and reaches a maximum ${\bar{\varepsilon }}_{M}$ of 5.34% (5.25%) in Ti−22Nb−4Ta (Ti−22Nb−2Al). It can be observed that the maximum recovery strain is about 3.89% in Ti−24Nb−3Al [4] and 3.20% in both Ti−22Nb−4Ta and Ti−22Nb−5Ta [7] at RT, respectively. For lower Al and Ta contents, the effect of solid solution strengthening plays a leading role in the increase in the recovered strain [4,44,69]. With increasing Al and Ta contents, the critical stress may become the dominant factor in the previous measurements for TiNb-based alloys [4,7,54,66,70,71]. From Figure 2d, it can be observed that both the predicted and measured ${\bar{\varepsilon }}_{M}$ of Ti−Nb−Al (except for Ti−22Nb−4Al) are higher than those of Ti−Nb−Ta, despite the different magnitudes of ${\bar{\varepsilon }}_{M}$ that appear in our 0 K calculations and RT measurements [4,7]. The deviation for Ti−Nb−Al may originate from the different compositions used in our calculations and available measurements [4] and intermetallic compounds or second-phase particles in the experiments [4,7].

**Figure 3:**
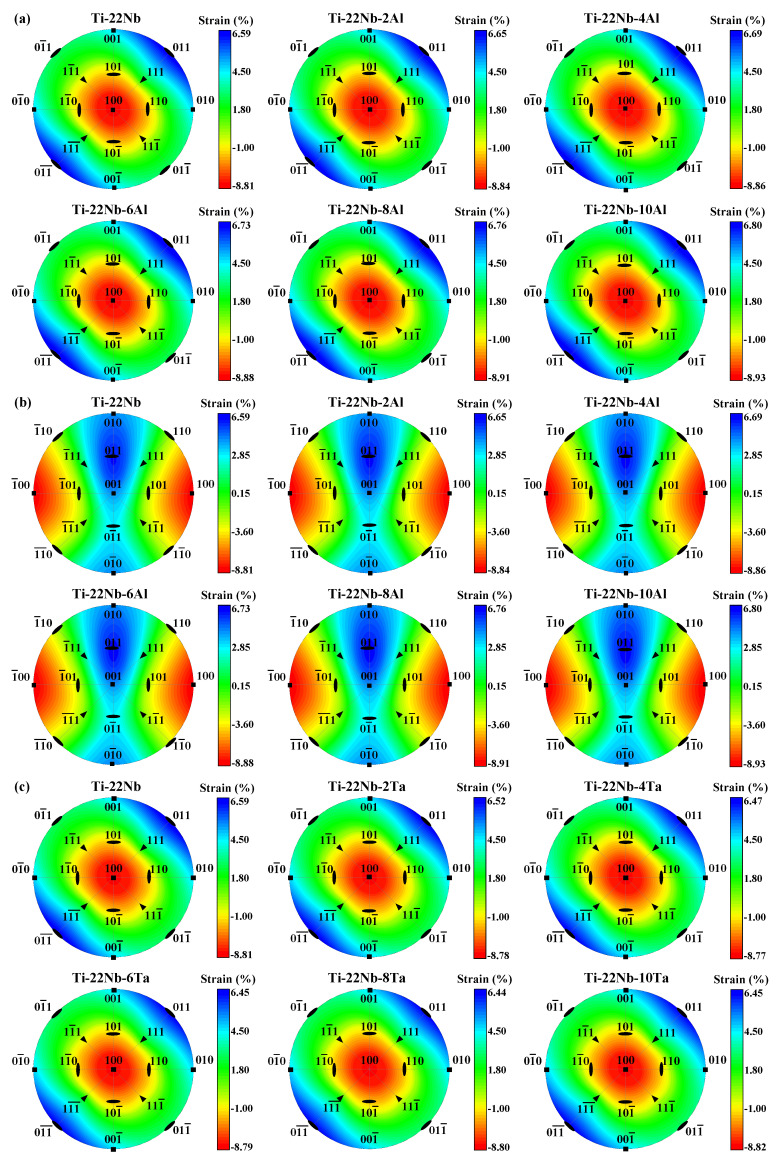

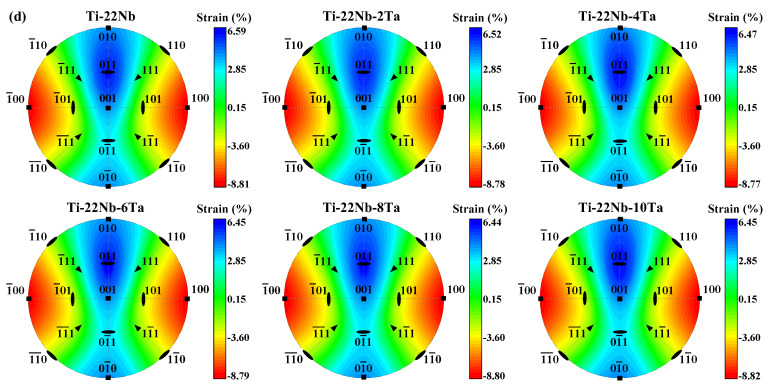
Stereographic projections of the lattice strains associated with the *β → α*″ phase transformation (**a**,**c**) along (100)*_β_* and (**b**,**d**) along (001)*_β_* for Ti−22Nb−*x*X (*x* = 0–10, in at. %; X = Al, Ta) from Equations (3) and (4). The largest elongation and the largest compression appear along ±[011]*_β_* and ±[100]*_β_*, respectively.

In Figure 3, we demonstrate the contour plots of the lattice strain (Equation (4)) using stereographic projections on the (100)*_β_* and (001)*_β_* of the *β* unit cell in Ti−Nb−Al and Ti−Nb−Ta ternary alloys. For the sake of simplicity, only 17 orientations are marked in Figure 3. Deviations from uniform coloring easily illustrate the direction and degree of deviatoric behavior. The red (blue) color of the contour plots denotes the maximum negative (positive) strain. The contour plots indicate the maximum transformation strains of the martensitic transformations in Ti−Nb−Al and Ti−Nb−Ta ternary alloys. From Figure 3, it can be distinctly observed that the largest elongations are along ±[011]*_β_* and that the largest compressions occur along ±[100]*_β_* for Ti−Nb−Al and Ti−Nb−Ta alloys, agreeing with the observations on Ti−Nb binary alloys [68]. As shown in Figure 3a,b, an increase in elongated lattice strain ranges from 6.59% to 6.80% as Al content increases, while an increase in contracted lattice strain ranges from 8.81% to 8.93% for Ti−Nb−Al alloys. Namely, the largest contraction and the largest elongation in Ti−Nb−Al alloys linearly increases by 0.02 and 0.07%/at. %, respectively. The situation becomes different for the Ti−Nb−Ta system. From Figure 3c,d, it can be observed that the largest contraction in the Ti−Nb−Ta alloys remains almost constant at around 8.80%, while the largest elongation decreases by 0.03%/at. % with increasing Ta content.

**Figure 4:**
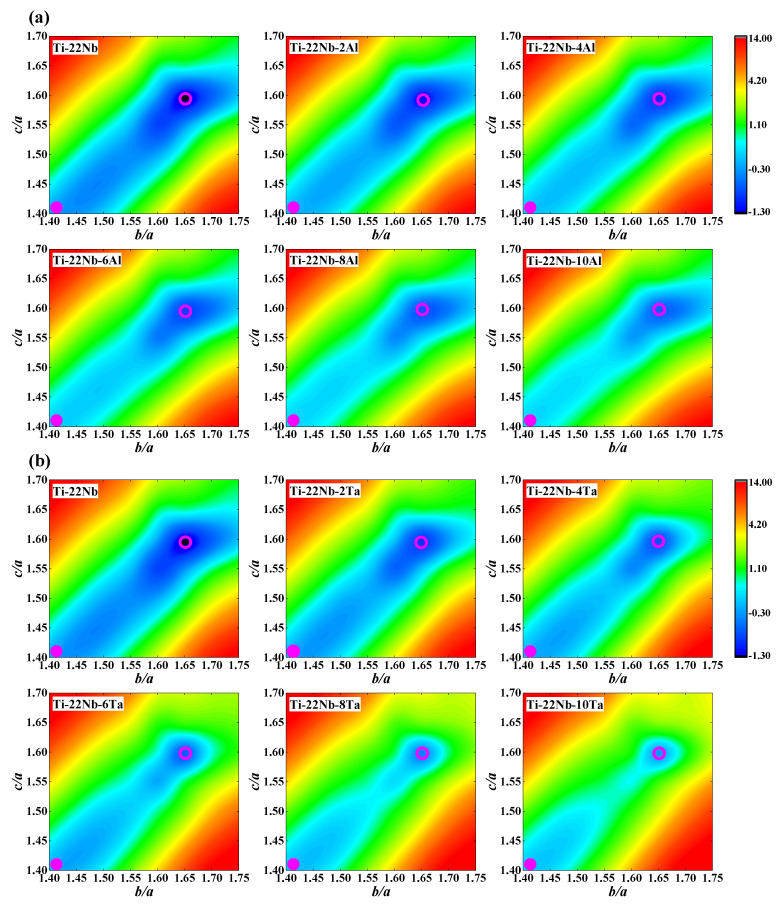
Total energy contours (in mRy) of the *β* and *α*″ phase transformation for (**a**) Ti−22Nb−*x*Al (*x* = 0–10, in at. %) and (**b**) Ti−22Nb−*x*Ta (*x* = 0–10, in at. %) ternary alloys as a function of the ratios of *b*/*a* and *c*/*a* from first-principles calculations. All energies are plotted relative to the corresponding *β* phase minima. The pink solid circles and pink open circles represent the *β* and *α*″ phases, respectively.

We calculate the total energies, *E*, at the corresponding equilibrium volume (Figure 1a) of the *β* phase in each composition. After fixing the shuffle *y*, the total energy contours of the *β* to *α*″ phase transformation for Ti−22Nb−*x*X (*x* = 0–10, in at. %; X = Al, Ta) are plotted in Figure 4 as a function of the ratios of *b*/*a* and *c*/*a*. From Figure 4, it can be seen that the most stable phase in the Ti−22Nb binary alloy appears to be the *α*″ phase (*c*/*a* = 1.60, *b*/*a* = 1.65), which is in line with the available experimental results [72,73,74] on Ti−Nb binaries. The *c*/*a* and *b*/*a* of the *α*″ phase (as shown in Table S1) for the Al-containing and Ta-containing ternaries remain almost unchanged, agreeing with the available measurement on Ti−Nb−Ta alloys [9]. Additionally, the predicted shuffle *y* (as shown in Table S1) for Ti−Nb−Ta ternary alloys is almost constant and is around 1.50, while the calculated *y* for Ti−Nb−Al alloys declines from 1.50 to 1.43 with increasing Al content. This finding indicates that Al has a greater ability to lower shuffle *y* than Ta, suggesting greater capacity on the lattice distortion induced by Al.

The energy difference, Δ*E_β→__α_*_″_ (Δ*E_β→__α_*_″_ *= E_α_*_″_ − *E_β_*, in mRy), between the *β* and *α*″ phases indicates the relative stability of the *β* and *α*″ phases. The Δ*E_β→__α_*_″_ < 0 shows that the *α*″ phase is more stable than the *β* phase. If the absolute value of the Δ*E_β→__α_*_″_ becomes smaller with increasing alloying elements, the relative stability of the *α*″ phase is regularly weakened and the ability to generate the *β* phase is gradually promoted. From Figure 4, it can be seen that the change in Δ*E_β→__α_*_″_ in the Ti−Nb−Al system is from −1.12 to −0.94 mRy with increasing Al content, revealing that the relative stability of the *α*″ phase weakly decreases. The Δ*E_β→__α_*_″_ of Ti−Nb−Ta alloys varies from −1.12 to −0.39 mRy with increasing Ta content, implying that the relative stability of the *α*″ phase strongly decreases. This finding demonstrates that Ta [7,75] is a much stronger *β* stabilizer in Ti alloys than Al [26], implying that Ta distinctly promotes the formation of the *β* phase when compared to doping Al. The available measurements have shown that both Nb and Al can act as *β* stabilizers in TiNb-based alloys [26,54,70,71]. From Figure 4, it can be observed that both Al and Ta can reduce energy differences in different magnitudes, but Ta shows a much stronger ability to stabilize the *β* phase than Al. Moreover, Al can reduce the energy difference between the *β* and *α*″ phases in Ti−Ta−Al ternary alloys [27].

For the sake of convenience, the martensitic transformation temperature is investigated based on a proposed hypothesis. In this work, we approximate the two Ti−Nb−Al and Ti−Nb−Ta ternaries into individual Ti−(Nb + Al) and Ti−(Nb + Ta) pseudobinaries, respectively. Since the measured *M*_s_ decreases by 40, 40, and 30 K with an increase of 1 at. % Nb, Al, and Ta, respectively, it is indicated that the doping of Nb [41], Al [4,5,6,54], and Ta [7] shows a similar magnitude order on the *M*_s_. Alternatively, it is found that the calculated Δ*E_β→__α_*_″_ for different Ti−Nb−Al alloys having the same (Nb + Al) content is almost the same (as shown in Figure S4). Namely, our calculated Δ*E_β→__α_*_″_ is insensitive to specific alloy components. Therefore, it is assumed that different Ti−Nb−Al alloys approximately possess the same Δ*E_β→__α_*_″_ if Ti−Nb−Al alloys contain the same (Nb + Al) content. Like Ti−Nb−Al, the Ti−Nb−Ta system having the same (Nb + Ta) content exhibits the same Δ*E_β→__α_*_″_. Consequently, Table 1 shows that Ti−Nb−X alloys containing the same (Nb + X) (X = Al, Ta) content have the same predicted *M*_s_ due to the same Δ*E_β→__α_*_″_ based on our pseudobinary hypothesis. For example, the Δ*E_β→α_*_″_ of both Ti−23Nb−3Al and Ti−24Nb−2Al is the same as that of Ti−22Nb−4Al since these three alloys contain the same (Nb + Al) content. Namely, they have the same Δ*E_β→α_*_″_ of −1.141 mRy and then possess the same predicted $M_{s}^{Al}$ of 335.2 K.

**Figure 5:**
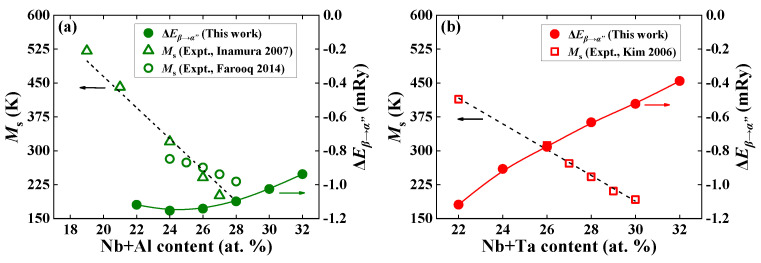
Theoretically calculated energy difference (Δ*E_β→α_*_″_, in mRy) for (**a**) Ti−22Nb−*x*Al (*x* = 0–10, in at. %) and (**b**) Ti−22Nb−*x*Ta (*x* = 0–10, in at. %) as a function of alloying elements, as well as the available measured martensitic transformation temperature (*M*_s_, in K) [4,6,7]. The measured alloys are Ti−(16, 18, 23, 24)Nb−3Al [6], Ti−24Nb−(0–4)Al [4], and Ti−22Nb−(4–8)Ta [7] ternary alloys. For completeness, Ti−22Nb [7] and Ti−24Nb [6] binary alloys are also displayed.

Figure 5a,b plot the available measured *M*_s_ [4,6,7] and the present theoretical Δ*E_β→__α_*_″_ for Ti−22Nb−*x*X (*x* = 0–10, X = Al, Ta) ternary alloys as functions of the (Nb + X) (X = Al, Ta) content. Note that the values of the Δ*E_β→__α_*_″_ are all negative, indicating that the *α*″ phase is more stable than the *β* phase. The absolute value of Δ*E_β→__α_*_″_ decreases with increasing Al and Ta contents, signifying that the relative stability of the *α*″ phase is gradually weakened and the tendency to generate the *β* phase is enhanced. This finding agrees with the available observations on Ti−Nb−Al [4] and Ti−Nb−Ta [7].

It is still a challenge to directly predict the *M*_s_ using a first-principles method. Based on former first-principles calculations [47,48], the lower the absolute Δ*E_β→__α_*_″_, the lower the *M*_s_. Furthermore, Minami et al. [47] and Sun et al. [48] correlated the *M*_s_ and the Δ*E_β→__α_*_″_ between the *β* and *α*″ phase for Ti−Nb binaries. Their correlations can qualitatively predict the composition dependence of the *M*_s_. However, the evaluated *M*_s_ derived from their empirical relationships [47,48] greatly overestimated the measurements overall. Despite the fact that the alloying effect on TiNb-based ternaries [47,48] and high-entropy alloys [48] has been qualitatively investigated, there is no quantitative research on TiNb-based ternary systems. Furthermore, extensive experimental observations have used different functions, such as linear [41,43,47], 1.5 degrees [45], and cubic polynomial [46], to fit the *M*_s_ for different Ti−Nb binary alloys. Therefore, these functions [41,43,45,46,47] used in binary systems may lower the accuracy of Ti-based ternary and multicomponent alloys due to the ignorance of the coupling effect of alloying elements. In this work, the coupling effect of alloying elements is considered by adopting a pseudobinary hypothesis on Ti−(Nb + Al) and Ti−(Nb + Ta) systems.

Here, we construct the relationships between the calculated composition-dependent Δ*E_β→__α_*_″_ for Ti−(Nb + Al) and Ti−(Nb + Ta) pseudobinaries with the measured *M*_s_. In this way, one may accurately determine the *M*_s_ employing first-principles calculations.

Since the alloying elements Al and Ta have different influences on the energy difference between the *β* and *α*″ phase, we separately fit two empirical relationships by connecting our theoretical Δ*E_β→__α_*_″_ with the measured *M*_s_ for Ti−Nb−Al [4,6] and Ti−Nb−Ta [7] alloys. For the Ti−Nb−Al system, an empirical relationship derived from Figure 5a can be expressed, as in Equation (5):

(5) MsAl=−8383×10∆Eβ→α″1.13+1154.3

For the Ti−Nb−Ta system, another empirical relationship draw from Figure 5b can be fitted, as in Equation (6):

(6) MsTa=−1119×10∆Eβ→α″4.36+1044.4

where the unit of $M_{s}^{Al}$ and $M_{s}^{Ta}$ is K, and the unit of Δ*E_β→__α_*_″_ is mRy. Although Al and Ta have similar alloying effects on the *M*_s_, their influences on the energy difference, Δ*E_β→__α_*_″_, are different. As shown in Equations (5) and (6), the different coefficients of Δ*E_β→__α_*_″_ for Al-containing and Ta-containing systems are 1.13 and 4.36, respectively. Based on the Δ*E_β→__α_*_″_ calculated from first-principles calculations, the theoretical *M*_s_ for Ti−Nb−Al and Ti−Nb−Ta alloys can be quickly predicted from Equations (5) and (6), respectively.

To assess the reliability of the predicted $M_{s}^{Al}$ by Equation (5) and $M_{s}^{Ta}$ by Equation (6), Table 1 displays the present predicted $M_{s}^{Al}$ and $M_{s}^{Ta}$, the available $M_{s}^{Expt,Al}$ and $M_{s}^{Expt,Ta}$ [4,6,7], and the estimated $M_{s}^{1}$ from former empirical relationships [48] for Ti−Nb−Al and Ti−Nb−Ta alloys. The composition dependence of the present predicted *M*_s_ for Ti−Nb−Al and Ti−Nb−Ta alloys reproduces their experimental counterparts. For Ti−Nb−Ta, the average error between the predicted $M_{s}^{Ta}$ by Equation (6) and available $M_{s}^{Expt,Ta}$ [7] is about 4%. Compared to the current $M_{s}^{Al}$ and $M_{s}^{Ta}$, the predicted $M_{s}^{1}$ by former empirical relationships [48] shows the unreasonable composition dependence of Ti−Nb−Al and Ti−Nb−Ta alloys and is greatly overestimated relative to the experimental counterparts [4,6,7]. When compared to the measurements [4,6], an opposite alloying effect of Al on the *M*_s_ can be estimated by fitting an empirical equation [45]. Therefore, the present empirical relationships of Equations (5) and (6) accurately predict the *M*_s_, corresponding to former empirical relationships [45,48]. As shown in Table 1, the error between $M_{s}^{Ta}$ and $M_{s}^{Expt,Ta}$ is relatively smaller than that of $M_{s}^{Al}$ and $M_{s}^{Expt,Al}$. The deviation in Equation (5) for Ti−Nb−Al may result from the different alloy compositions used in our calculated Δ*E_β→__α_*_″_ and the measured $M_{s}^{Expt,Al}$ [4,6]. The prediction of Equation (5) may further deteriorate for higher (Nb + Al) contents, such as the predicted $M_{s}^{Al}$ of −85.5 K for Ti−22Nb−10Al.

**Figure 6:**
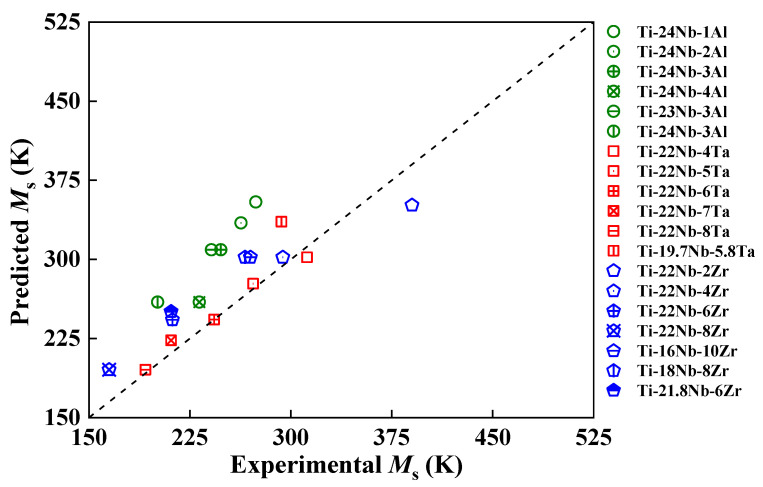
Comparison between present predicted and available experimental [4,6,7,12,13] martensitic transformation temperature (*M*_s_, in K) for Ti−Nb−Al (green circles), Ti−Nb−Ta (red squares), and Ti−Nb−Zr (blue pentagons) ternary alloys. The estimated *M*_s_ in Ti−Nb−Al is from Equation (5), while the predicted *M*_s_ in Ti−Nb−Ta and Ti−Nb−Zr is from Equation (6).

As shown in Table 1 and Figure 6, both the present predictions and available measurements [4,6,7] qualitatively predict the similar composition dependence of *M*_s_, despite the fact that the predicted $M_{s}^{Al}$ and $M_{s}^{Ta}$ are somehow higher than the relative $M_{s}^{Expt,Al}$ and $M_{s}^{Expt,Ta}$. The predicted *M*_s_ decreases by 28 and 30 K with an increase of 1 at. % Al and Ta, corresponding to a decrease in the measured *M*_s_ by 13 [4] and 30 K [7], respectively. Both $M_{s}^{Ta}$ and $M_{s}^{Expt,Ta}$ begin to fall below RT when *x* > 4 at. % Ta. The case is quite complex for Ti−Nb−Al. The predicted $M_{s}^{Al}$ for Ti−22Nb−*x*Al starts to fall below RT if *x* > 6 at. % Al. However, the $M_{s}^{Al}$ for Ti−23Nb−*x*Al and Ti−24Nb−*x*Al (except for Ti−24Nb−4Al) are above RT, while the $M_{s}^{Expt,Al}$ [4,6] are below RT. However, the predicted $M_{s}^{Al}$ decreases by 46 K/1 at. % Nb for Ti−(23–24)Nb−3Al alloys, which is consistent with the measured decline of 40 K and 40 K for Ti−Nb binary alloys [41] and Ti−Nb−Al ternary alloys [6], respectively. This finding suggests that the coupling effect of alloying elements are appropriately described based on our pseudobinary hypothesis.

To further directly compare the discrepancy between our predicted and measured *M*_s_, in Figure 6, we plot the predicted *M*_s_ for Ti−Nb−Al by Equation (5) and for Ti−Nb−Ta and Ti−Nb−Zr by Equation (6), along with the measured *M*_s_ for Ti−Nb−Al [4,6], Ti−Nb−Ta [7,13], and Ti−Nb−Zr [12,13,44] ternary alloys. Since Zr and Ta have similar alloying effects on the *M*_s_, we assume that the energy difference, Δ*E_β→__α_*_″_, in the Ti−Nb−Zr alloy is approximate to the Δ*E_β→__α_*_″_ in the Ti−Nb−Ta alloy when the (Nb + Zr) content is equal to the (Nb + Ta) content. As shown in Figure 6, there are average errors of about 28%, 4%, and 13% between our predicted and measured *M*_s_ for Ti−Nb−Al [4,6], Ti−Nb−Ta [7,13], and Ti−Nb−Zr [12,13,44], respectively. It can be concluded that there is a general agreement between the prediction and measurements.

Ren and Otsuka [76] explained the compositional dependence of *M*_s_ using the Landau-type model. In the process of martensitic transformation, elastic modulus decreases gradually with cooling and reaches a critical value before martensitic transformation [76]. Therefore, if the elastic constants *C*′ and *C*_44_ of the *β* phase increase, the cooling should continue to lower temperatures before a critical elastic constant and *M*_s_ decreases. The critical elastic constraint of martensite alloys is temperature-independent. Therefore, when the elastic modulus changes, the *M*_s_ must also change due to critical elastic constraints. The relationship [76] between the *M*_s_ and elastic modulus (*C*) can be approximately expressed as follows:

(7) $$\frac{dM_{s}}{dC}=-\frac{1}{\gamma C}$$

where the *M*_s_ is the martensitic transformation temperature, *C* is the elastic modulus (the *C* can be either *C*_44_, *C*′, or some other elastic modulus), and *γ* is the temperature coefficient of elastic modulus. Therefore, the increase in the martensite temperature is consistent with the decrease in elastic modulus. The relationship proposed by Ren and Otsuka [76] has been widely accepted for investigating the *M*_s_ for TiNi-based [50,77,78], Cu−Al−Mn [79], and Ni-based [49,80,81,82] systems. These studies [49,50,77,78,79,80,81,82] have treated elastic modulus as one of the indicators to predict martensite temperature. For example, Cao et al. [81] used *C*′ to evaluate the *M*_s_, indicating that a large *C*′ would inhibit martensite transformation. That is, besides the energy difference, Δ*E_β→α_*_″_, the elastic moduli, *C*′ and *C*_44_, can be considered as predictors of the *M*_s_ [49,50], implying a correlation between the elastic moduli (*C*′ and *C*_44_) and the *M*_s_.

**Figure 7:**
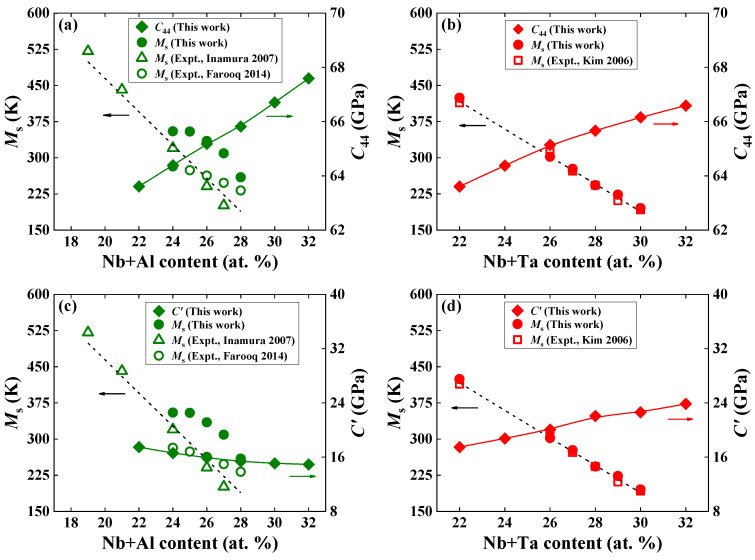
Theoretically estimated martensitic transformation temperature (*M*_s_, in K) and elastic moduli, *C*_44_ and *C*′, (in GPa) for (**a**,**c**) Ti−22Nb−*x*Al (*x* = 0–10, in at. %) and (**b**,**d**) Ti−22Nb−*x*Ta (*x* = 0–10, in at. %) ternary alloys. The available measured *M*_s_ for Ti-Nb-Al [4,6] and Ti-Nb-Ta [7] ternary alloys are shown for comparison. Note that the available measured alloy compositions are Ti−(16, 18, 23, 24)Nb−3Al [6] and Ti−24Nb−(1–4)Al [4] for Ti-Nb-Al alloys and Ti−22Nb−(4–8)Ta [7] for Ti−Nb−Ta alloys. For completeness, the *M*_s_ of Ti−22Nb [7] and Ti−24Nb [4] are also displayed. Note that different colored arrows in the figure mark the *M*_s_, *C*_44_, or *C*′, respectively.

In Figure 7, we display the calculated *C*′ and *C*_44_ and the predicted $M_{s}^{Al}$ and $M_{s}^{Ta}$, as well as the available experimental $M_{s}^{Expt,Al}$ [4,6] and $M_{s}^{Expt,Ta}$ [7] as a function of alloying elements. As for Ti−Nb−Al ternary alloys, *C*_44_ (*C*′) increases (decreases) with increasing Al content, corresponding to a decrease in $M_{s}^{Al}$ and $M_{s}^{Expt,Al}$. For Ti−Nb−Ta ternary alloys, both *C*_44_ and *C*′ increase, whereas $M_{s}^{Ta}$ and $M_{s}^{Expt,Ta}$ decrease with increasing Ta content. From Figure 7a,b, it can be observed that for both Ti−Nb−Al and Ti−Nb−Ta alloys, the larger the calculated *C*_44_, the lower the *M*_s_. There is a similar relationship between the calculated *C*′ and $M_{s}^{Ta}$ and $M_{s}^{Expt,Ta}$ for the Ti−Nb−Ta system in Figure 7d. However, such a relationship connecting *C*′ with *M*_s_ is invalid for Ti−Nb−Al, as shown in Figure 7c. This finding implies that the elastic modulus, *C*_44_, may be regarded as a prefactor of the composition dependence of the *M*_s_.

## 4. Conclusions

Using first-principles EMTO-CPA calculations, we systematically calculated the total energy contours, lattice deformation strains (*η*_1_, *η*_2_, and *η*_3_), maximum transformation strains ($\varepsilon_{M}^{i}$), and the martensitic transition temperature (*M*_s_) during the *β → α*″ phase transformation for two selected Ti−Nb−Al and Ti−Nb−Ta ternary alloys. The present theoretical calculations and the available experiments gave the same composition dependence on the lattice strains and *M*_s_. As for the calculated stereographic projections of lattice strains alongside phase transformation along (100)*_β_* and (001)*_β_*, the largest elongation and the largest contraction due to the lattice strain occurred along ±[011]*_β_* and ±[100]*_β_*, respectively. The addition of Al and Ta increased and decreased the transformation strain by 0.07 and 0.03%/at. %, respectively.

The effect of either Al or Ta additions on the energy difference (Δ*E_β→__α_*_″_) between the *β* and *α*″ phases was also studied, suggesting that both Al and Ta can lower Δ*E_β→__α_*_″_. The relative phase stability of *α*″ gradually weakened but the tendency to generate the *β* phase became stronger as Al and Ta contents increased. Aiming to directly assess the *M*_s_ from first-principles calculations, two empirical relationships were fitted by associating the measured *M*_s_ with the calculated Δ*E_β→__α_*_″_. When compared to the overestimation by the existing relationships, there was a satisfactory agreement between the predicted and measured *M*_s_, implying that the proposed relationships could accurately describe the coupling effect of alloying elements on the *M*_s_. In this work, the theoretically predicted *M*_s_ were reduced by around 46, 28, and 30 K with an increase of 1 at. % Nb, Al, and Ta, respectively, corresponding to measured declines in *M*_s_ by 40, 40, and 30 K, respectively. Moreover, there was a correlation between *M*_s_ and *C*_44_, implying that an elastic modulus can be used as a prefactor to evaluate composition-dependent *M*_s_. This work can contribute to accurately estimating the *M*_s_ of Ti-based alloys.

## Figures and Tables

**Table 1:** Theoretically calculated energy difference (Δ*E_β→__α_*_″_, in mRy) between the *β* and *α*″ phases and estimated martensitic transformation temperature (*M*_s_, in K) for Ti−Nb−Al and Ti−Nb−Ta (in at. %) alloys. Note that the predicted $M_{s}^{Al}$ and $M_{s}^{Ta}$ are derived from Equations (5) and (6), respectively. For comparison, we show the available experimental $M_{s}^{Expt,Al}$ [4,6] and $M_{s}^{Expt,Ta}$ [7] and the evaluated $M_{s}^{1}$ from former empirical relationships [48].

(a) Ti−Nb−Al	Δ*E_β→α_*_″_	$M_{s}^{Al}$	$M_{s}^{1}$ [48]	$M_{s}^{Expt,Al}$
Ti−22Nb−2Al	−1.153	354.8	625.4	-
Ti−22Nb−4Al	−1.141	335.2	623.2	-
Ti−22Nb−6Al	−1.098	260.3	615.2	-
Ti−22Nb−8Al	−1.025	116.9	601.1	-
Ti−22Nb−10Al	−0.938	−85.5	583.5	-
Ti−23Nb−3Al	−1.141	335.2	623.2	241 [6]
Ti−24Nb−3Al	−1.126	309.1	620.4	201 [6]
Ti−24Nb-1Al	−1.147	344.5	624.3	274 [4]
Ti−24Nb−2Al	−1.141	335.2	623.2	263 [4]
Ti−24Nb−3Al	−1.126	309.1	620.4	248 [4]
Ti−24Nb−4Al	−1.098	260.3	615.1	232 [4]
(b) Ti−Nb−Ta	Δ*E_β→α_*_″_	$M_{s}^{Ta}$	$M_{s}^{1}$ [48]	$M_{s}^{Expt,Ta}$
Ti−22Nb−2Ta	−0.907	351.4	577.1	-
Ti−22Nb−4Ta	−0.777	302.0	548.9	312 [7]
Ti−22Nb−5Ta	−0.714	276.9	534.5	272 [7]
Ti−22Nb−6Ta	−0.632	242.9	514.6	243 [7]
Ti−22Nb−7Ta	−0.586	223.2	503.2	211 [7]
Ti−22Nb−8Ta	−0.523	195.5	486.7	192 [7]
Ti−22Nb−10Ta	−0.388	132.6	448.1	-

## Data Availability

The research data are available on request from the corresponding author.

## Supplementary Materials

The following supporting information can be downloaded at: https://www.mdpi.com/article/10.3390/ma16176069/s1. Figure S1. The crystal structures and the lattice correspondence of the *β*, *α*″ and *α* phase; Figure S2. The relationship of the lattice deformation strains (*η*_1_, *η*_2_, and *η*_3_), the maximum transformation strain ($\varepsilon_{M}^{i}$), and the average maximum transformation strain (${\bar{\varepsilon }}_{M}$); Figure S3. Schematic diagram for selected representative orientations of stereographic projections of the lattice strains associated with the *β → α*″ phase transformation; Figure S4. The calculated energy difference Δ*E_β__→__α_*_″_ for Ti−(0−40)Nb−(0−20)Al alloys; Table S1. Predicted *b/a*, *c/a* and shuffle *y* of Ti−22Nb−*x*X (*x* = 0, 2, 4, 6, 8, 10, in at. %; X = Al, Ta) alloys. References [83,84] can be found in Supplementary Materials.
