# Supplementary material for: Alloying Effect on Transformation Strain and Martensitic Transformation Temperature of Ti-Based Alloys from Ab Initio Calculations
Source: Materials (Basel). 2023 Sep 4;16(17):6069. doi: 10.3390/ma16176069 (PMC10488558; doi:10.3390/ma16176069)
Supplement: Supplementary file 1 [file materials-16-06069-s001.zip › materials-2563109-supplementary.pdf]

Alloying effect on transformation strain and martensitic transformation temperature of Ti-based alloys from ab initio calculations

Honglin Fang[1#], Xingge Xu[1#], Hualei Zhang[1,2*], Qiaoyan Sun[1*], Jun Sun[1]

[1]State Key Laboratory for Mechanical Behavior of Materials, Frontier Institute of Science and Technology, Xi'an Jiaotong University, Xi'an, 710049, China

[2]Key Laboratory of Shaanxi Province for Craniofacial Precision Medicine Research, College of Stomatology, Xi'an Jiaotong University, Xi'an, 710049, China

This PDF file includes:

Supplemental text

Figs. S1 to S4

Table S1

References

* Corresponding authors.
E-mail addresses: hualei@xjtu.edu.cn; qysun@mail.xjtu.edu.cn;
# These authors contributed equally.

Figure S1 shows the crystallographic relationship among the β, α'', and α phases [1−3]. The procedure of $\beta \rightarrow \alpha''$ phase transformation can be described as a shear by $\{211\}\langle 111\rangle_\beta$ and a shuffle by $\{011\}\langle 0\bar{1}1\rangle_\beta$ [4]. The crystalline positions of atoms in the α'' phase can be described as (0, 0, 0), (1/2, 1/2, 0), (1/2, y, 1/2), and (0, 1/2+y, 1/2). The shuffle y varies upon different alloying elements and compositions. The lattice constants of the α'' phase change due to the $\{211\}\langle 111\rangle_\beta$ shear, therefore the b/a and c/a ratios also relate to the shear. The α'' phase is equivalent to the β phase when b/a = c/a = $\sqrt{2}$ and shuffle y = 0, while the α'' phase becomes to α phase when b/a = $\sqrt{3}$ and y/b = 1/6. The α'' phase can be regarded as an intermediate phase in the process of $\beta \rightarrow \alpha$ transformation [4, 5].

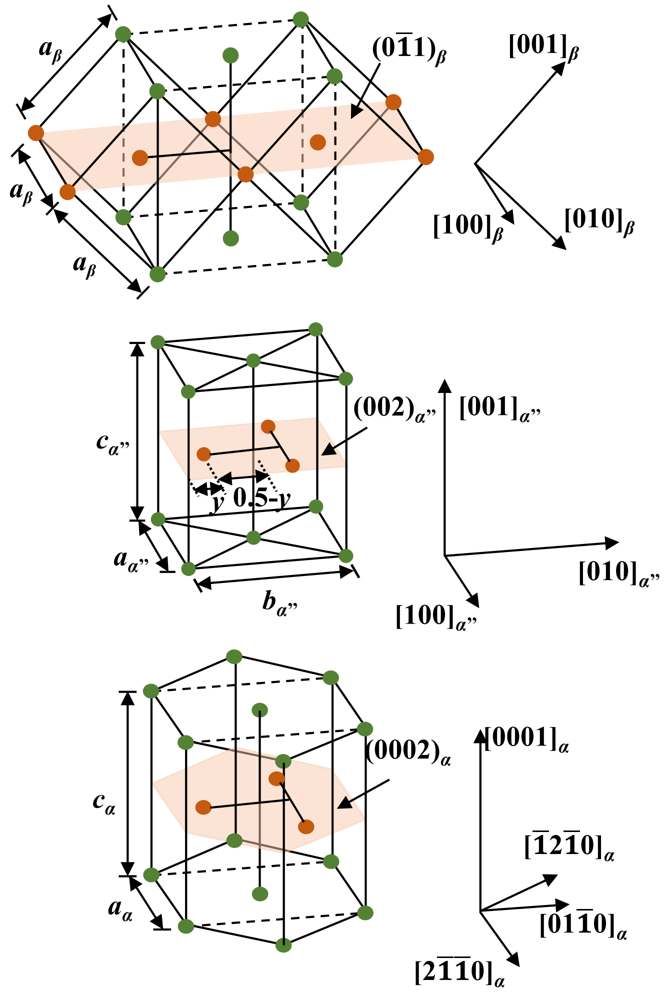

Figure S1. The crystal structures and the lattice correspondence of the β, α'', and α phase. The shuffle y is on the $\{011\}_\beta$ plane (in orange).

The lattice deformation strains η_1, η_2, and η_3 required to form the α'' phase to the β phase are along the three principal axes of $[100]_\beta$, $[011]_\beta$, and $[0\bar{1}1]_\beta$, respectively.

The maximum transformation strains $\varepsilon_{\mathrm{M}}^{i}$ are along a certain orientation $\vec{x}$ in the β phase. During the $\beta \rightarrow \alpha''$ martensitic transition, the vector $\vec{x}$ in the β phase is transformed to $\vec{x'}$ in the α'' phase due to the lattice distortion. Thus, the maximum transformation strain $\varepsilon_{\mathrm{M}}^{i}$ can be calculated by the lattice distortion matrix **T**.

The average maximum strains $\bar{\varepsilon}_{\mathrm{M}}$ are calculated by spline interpolation of the maximum transformation strain $\varepsilon_{\mathrm{M}}^{i}$ along 13 representative orientations located in the [001]−[011]−[111] standard stereographic triangle (shown in the Figure S3).

(a) Lattice deformation strains

$$\begin{cases} \eta_1 = \dfrac{a' - a_\beta}{a_\beta} & [100]_\beta \\ \eta_2 = \dfrac{b' - \sqrt{2}a_\beta}{\sqrt{2}a_\beta} & [011]_\beta \\ \eta_3 = \dfrac{c' - \sqrt{2}a_\beta}{\sqrt{2}a_\beta} & [0\bar{1}1]_\beta \end{cases}$$

(b) Maximum transformation strains

$$\varepsilon_{\mathrm{M}}^{i} = \frac{|\vec{x'}| - |\vec{x}|}{|\vec{x}|}, \quad \vec{x'} = \mathbf{T}\vec{x}. \quad T = \begin{bmatrix} \dfrac{a'}{a_\beta} & 0 & 0 \\ 0 & \dfrac{b'+c'}{2\sqrt{2}a_\beta} & \dfrac{b'-c'}{2\sqrt{2}a_\beta} \\ 0 & \dfrac{b'-c'}{2\sqrt{2}a_\beta} & \dfrac{b'+c'}{2\sqrt{2}a_\beta} \end{bmatrix}$$

(c) Average maximum transformation strains $\bar{\varepsilon}_M$ can be extracted from the spline interpolation of the maximum transformation strains ε_M^i along 13 representative orientations.

Figure S2. The relationship among (a) lattice deformation strains (η_1, η_2, and η_3), (b) the maximum transformation strains ($\varepsilon_{\mathrm{M}}^{i}$), and (c) the average maximum transformation strains ($\bar{\varepsilon}_{\mathrm{M}}$).

In Figure S3a, we used the stereographic projections of lattice strains on $(001)_\beta$ to display the 57 representative orientations. The orientation selection method is to divide each line segment shown in Figure S3a into eight parts. The selected orientations represent the vertex and the midpoint of the edge at each standard stereographic triangle. Figure S3b displays the [100]−[110]−[111] standard stereographic triangle and plots 13 representative orientations to divide $\widehat{100-111}$, $\widehat{100-221}$, and $\widehat{100-110}$ into

four equal parts. The orientation selection method can make the image more accurate by evenly dividing the stereographic projections.

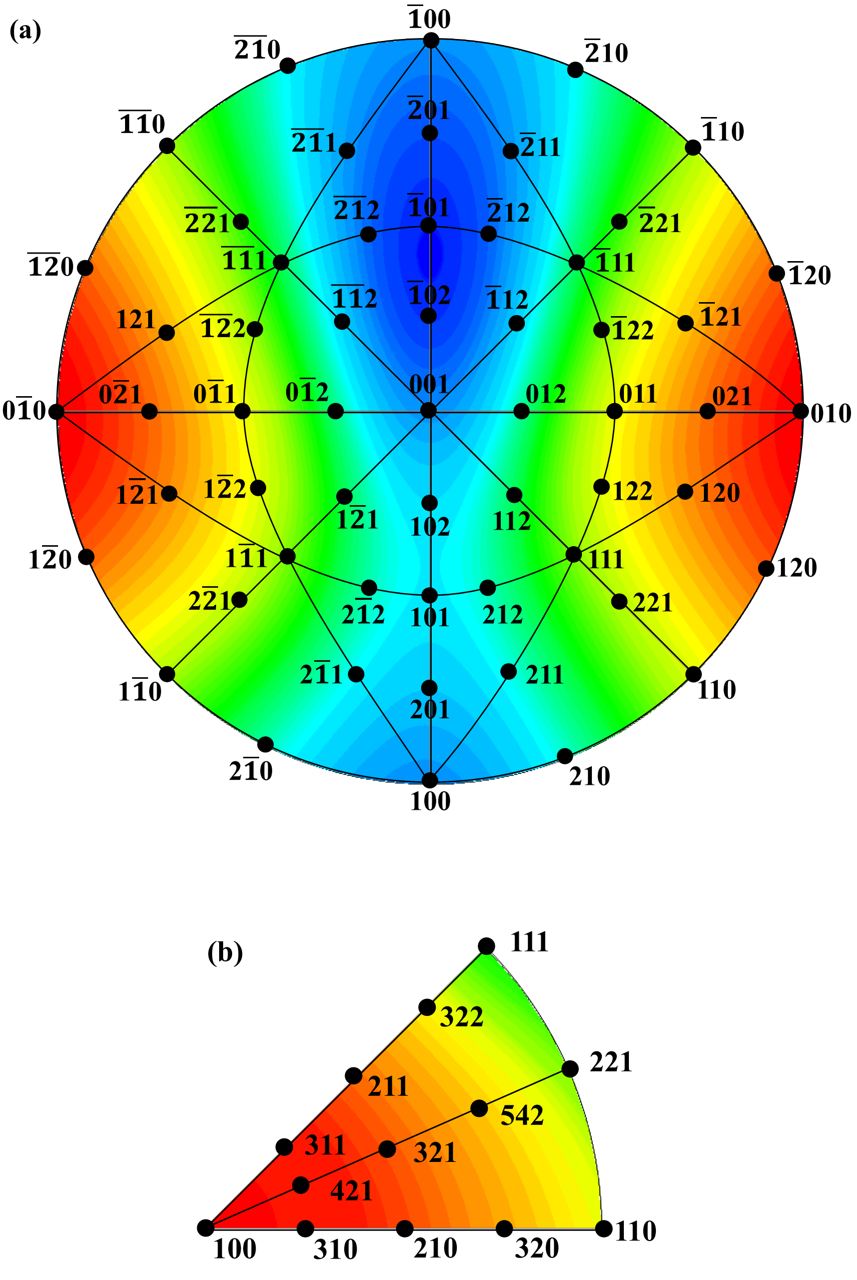

Figure S3. Schematic diagram for selected representative orientations of stereographic projections of the lattice strains associated with the $\beta \rightarrow \alpha''$ phase transformation. The positions of (a) 57 representative crystallographic orientations on $(011)_\beta$ and (b) 13 representative orientations located in the [100]−[110]−[111] standard stereographic triangle have been marked in the figure.

Table S1 displays that the *c/a* and *b/a* of α'' phase for the Ti−Nb−Al and Ti−Nb−Ta

ternaries have no evident change, in line with the available measurement in Ti−Nb−Ta alloys [3]. Furthermore, the predicted shuffle y for Ti−Nb−Ta ternary alloys keeps almost constant and is around 1.50, while the calculated y for Ti−Nb−Al alloys decreases from 1.50 to 1.43 with doping Al. As Table S1 shows, Al has greater influence on decreasing shuffle y than Ta. The possible reason is that Al can cause more lattice distortion than Ta in TiNb-based alloys due to the atomic radius r(Ta) > r(Al).

Table S1. Predicted b/a, c/a and shuffle y of Ti−22Nb−xX (x = 0, 2, 4, 6, 8, 10, in at. %; X = Al, Ta) alloys

Alloys	b/a	c/a	shuffle y
Ti−22Nb	1.65	1.60	0.150
Ti−22Nb−2Al	1.65	1.60	0.149
Ti−22Nb−4Al	1.66	1.60	0.148
Ti−22Nb−6Al	1.66	1.60	0.147
Ti−22Nb−8Al	1.66	1.60	0.145
Ti−22Nb−10Al	1.66	1.60	0.144
Ti−22Nb	1.65	1.60	0.150
Ti−22Nb−2Ta	1.65	1.60	0.150
Ti−22Nb−4Ta	1.65	1.60	0.149
Ti−22Nb−6Ta	1.65	1.60	0.151
Ti−22Nb−8Ta	1.65	1.60	0.149
Ti−22Nb−10Ta	1.65	1.60	0.149

Based on our first-principles calculations, for Ti−(0−40)Nb−(0−20)Al alloys, the absolute value of energy difference $\Delta E_{\beta\rightarrow\alpha''}$ increases with alloying Al and Nb. In Figure S4, except the compositions for which the $\beta \rightarrow \alpha''$ phase transformation has great difficulty to happen (purple balls), alloys with the same (Nb+Al) (red balls) appear the same color range of $\Delta E_{\beta\rightarrow\alpha''}$. Note that Al shows certain characteristics of β-stable

element in Ti alloys containing more β-stable elements, which seems to be largely dependent on other elements in the alloys [6−8]. Therefore, Al cannot appear the ability to stable the β phase for alloys with less Nb content. For example, Ti−10Nb−15Al and Ti−20Nb−5Al share the same (Nb+Al) content, but do not possess the same $\Delta E_{\beta\rightarrow\alpha''}$. Here we mark the most evident alloys Ti−(25−x_1)Nb−x_1Al and Ti−(24−x_2)Nb−x_2Al in the black dashed circle. Thus, it can be supposed that Ti−Nb−Al alloys with the same (Nb+Al) share the same $\Delta E_{\beta\rightarrow\alpha''}$.

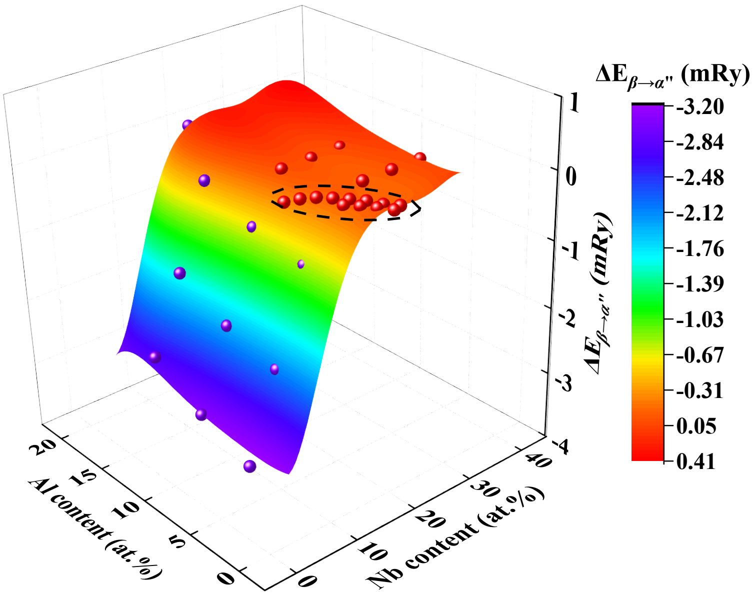

Figure S4. The calculated energy difference $\Delta E_{\beta\rightarrow\alpha''}$ for Ti−(0−40)Nb−(0−20)Al alloys. The purple balls represent Ti−5Nb−(5, 10, 15)Al, Ti−10Nb−(5, 10, 15)Al, and Ti−15Nb−(5, 10, 15)Al. The red balls represent Ti−(25−x_1)Nb−x_1Al (x_1 = 1−8), Ti−(24−x_2)Nb−x_2Al (x_2 = 1−4), Ti−20Nb−(10, 15)Al, Ti−25Nb−(5, 10, 15)Al, Ti−30Nb−(5, 10)Al, and Ti−35Nb−5Al. The marked area is for Ti−(25−x_1)Nb−x_1Al and Ti−(24−x_2)Nb−x_2Al alloys (x_1 = 1−8, x_2 = 1−4).

Acknowledgements

H. Fang and X. Xu contributed equally to this work. The National Natural Science Foundation of China (No.51871175 and 51671158), Opening Project (No.2021LHM-KFKT005) of Key Laboratory of Shaanxi Province for Craniofacial Precision Medicine

Research, and the 111 project 2.0 (No. BP0618008) and are acknowledged for financial support.

References

[1] X. Sun, H.L. Zhang, D. Wang, Q.Y. Sun, S.S. Zhao, S. Lu, W. Li, L. Vitos, X.D. Ding, Large recoverable strain with suitable transition temperature in TiNb-based multicomponent shape memory alloys: First-principles calculations, Acta Materialia **221** (2021) 117366.

[2] H.Y. Kim, Y. Ikehara, J.I. Kim, H. Hosoda, S. Miyazaki, Martensitic transformation, shape memory effect and superelasticity of Ti−Nb binary alloys, Acta Materialia **54** (2006) 2419.

[3] H.Y. Kim, J. Fu, H. Tobe, J.I. Kim, S. Miyazaki, Crystal Structure, Transformation Strain, and Superelastic Property of Ti−Nb−Zr and Ti−Nb−Ta Alloys, Shape Memory and Superelasticity **1** (2015) 107.

[4] Q. Liang, D. Wang, Y. Zheng, S. Zhao, Y. Gao, Y. Hao, R. Yang, D. Banerjee, H.L. Fraser, Y. Wang, Shuffle-nanodomain regulated strain glass transition in Ti−24Nb−4Zr−8Sn alloy, Acta Materialia **186** (2020) 415.

[5] C.X. Li, H.B. Luo, Q.M. Hu, R. Yang, F.X. Yin, O. Umezawa, L. Vitos, Lattice parameters and relative stability of α'' phase in binary titanium alloys from first-principles calculations, Solid State Communications **159** (2013) 70.

[6] M. Abdel-Hady, H. Fuwa, K. Hinoshita, H. Kimura, Y. Shinzato, M. Morinaga, Phase stability change with Zr content in β-type Ti–Nb alloys, Scripta Materialia **57** (2007) 1000.

[7] F. Zhang, Z.G. Yu, C.Y. Xiong, W.T. Qu, B.F. Yuan, Z.G. Wang, Y. Li, Martensitic transformations and the shape memory effect in Ti−Zr−Nb−Al high-temperature shape memory alloys, Materials Science and Engineering: A **679** (2017) 14.

[8] T.T. Yao, Y.G. Zhang, L. Yang, Z.Q. Bu, J.F. Li, A metastable Ti−Zr−Nb−Al multi-principal-element alloy with high tensile strength and ductility, Materials Science and Engineering: A **851** (2022) 143646.
